# Chronic Rhinosinusitis Management: A Narrative Review Comparing Interventional Treatment With Osteopathic Manipulation

**DOI:** 10.7759/cureus.70276

**Published:** 2024-09-26

**Authors:** Casey R Klover, Vasavi R Gorantla

**Affiliations:** 1 Surgery, West Virginia School of Osteopathic Medicine, Lewisburg, USA; 2 Medical Education, California University of Science and Medicine, Colton, USA

**Keywords:** chronic rhinosinusitis, chronic rhinosinusitis with nasal polyps, chronic rhinosinusitis without nasal polyps, functional endoscopic sinus surgery, osteopathic manipulation

## Abstract

Chronic rhinosinusitis (CRS) is an inflammatory disease affecting the nasal cavity and paranasal sinuses. Diagnosis of CRS typically requires two of the following four symptoms to persist for a minimum of 12 weeks: anterior or posterior nasal drainage, hyposmia or anosmia, facial pain or pressure, or nasal obstruction. Additionally, diagnosis necessitates the visualization of inflammation on physical examination or diagnostic imaging. This review aims to compare the outcomes of CRS patients who undergo interventional treatment via functional endoscopic sinus surgery (FESS) to those who opt for noninterventional treatment with osteopathic manipulation. FESS is not the sole interventional method utilized for CRS, but it is the focus of this review since it is the gold standard surgical treatment, encompassing a variety of techniques. Although there is limited literature discussing the use of osteopathic manipulative treatment (OMT) to remedy CRS, some evidence indicates OMT can alleviate symptoms for individuals seeking non-surgical alternatives. Moreover, osteopathic manipulation for CRS may be beneficial for patients unresponsive to previous medical or surgical management. Pharmacologic treatment is typically the initial approach to CRS and is discussed briefly herein, though it largely falls beyond the scope of this review.

## Introduction and background

The clinical practice guidelines accepted by the American Academy of Otolaryngology-Head and Neck Surgery determine the diagnostic criteria for chronic rhinosinusitis (CRS) to include the presence of two of the following four symptoms lasting for at least 12 weeks: anterior or posterior nasal drainage, hyposmia or anosmia, facial pain or pressure, or nasal obstruction. Diagnosis also requires the presence of inflammation on physical examination or diagnostic imaging [1].

The prevalence of CRS varies significantly across epidemiologic studies, but one study estimates that it affects between 5% and 12% of the global population [2]. In congruence with these data, it was found that over 10% of the US population meet the symptomatic criteria for CRS for a minimum of 12 weeks at any given time; however, this estimation excludes the objective evidence of inflammation required for accurate diagnosis [1,3]. There is considerable debate regarding the true prevalence of CRS, complicated by evolving diagnostic guidelines and the frequent conflation with recurrent acute rhinosinusitis due to the similarity in symptomatology [2,4]. In 2018, approximately 2.7 million patients in the United States were given a primary diagnosis of CRS in physician offices, with an annual estimated direct cost between $10 and $13 billion, underscoring the significant impact of CRS on the US population [5,6].

CRS is believed to be a heterogeneous disease with a pathogenesis impacted by both genetic and environmental factors [7,8]. Examples of possible predisposing conditions include asthma, cystic fibrosis, ciliary dyskinesia, chronic obstructive pulmonary disease, anatomic variation, an immunocompromised state, and allergies [1,9-14]. There is also evidence that suggests females develop CRS more often than males in all age groups [15]. CRS is mainly categorized based on the absence or presence of nasal polyps, which are pedunculated lesions typically located in the middle meatus [2,8]. CRS without nasal polyps (CRSsNP) is strongly associated with facial pain or pressure and rhinorrhea, whereas CRS with nasal polyps (CRSwNP) is strongly associated with hyposmia and nasal obstruction [8]. Evidence from electronic health records suggests that CRSsNP is more prevalent than CRSwNP in all age groups and declines after age 65, while diagnosis of CRSwNP remains constant with age [16-18].

Treatment protocols for CRS exhibit notable variation, with some clinical guidelines influenced by the presence of polyps. First-line treatment typically includes intranasal corticosteroids and nasal saline irrigation, with the optional addition of antibiotics, systemic steroids, antihistamines, decongestants, or leukotriene modifiers. Although this is not an exhaustive list, it does encompass the most utilized pharmacologic therapies [1,19,20]. When these treatment options prove ineffective, patients may pursue functional endoscopic sinus surgery (FESS) as a more invasive option. The objective of this interventional method is to use an endoscope to surgically enlarge the sinuses to restore proper drainage and ventilation [21]. However, some patients opt for osteopathic manipulative treatment (OMT) as an alternative to surgery or if the surgical outcome was undesirable. OMT begins with a physician diagnosing somatic dysfunction using palpatory skills, followed by physical manipulation to aid in the restoration of homeostasis [22]. This review aims to compare the effectiveness of interventional treatment via FESS to noninterventional treatment via OMT in the management of CRS.

## Review

Assessment tools for CRS

The sino-nasal outcome test (SNOT) is a widely utilized tool to assess the sinonasal health of patients. Initially named the Rhinosinusitis Outcome Measure-31 (RSOM-31), this test has evolved into four newer iterations distinguished by the number of questions they contain: SNOT-16, SNOT-20, SNOT-22, and SNOT-25. SNOT assessments are frequently administered to individuals with CRS to evaluate how the condition impacts their physical and emotional well-being. Each question prompts respondents to rank the severity of a specific symptom or emotion related to their sinonasal health from zero to five for the past two weeks, with five indicating the highest severity. Patients can also identify the symptoms and emotions of the highest significance to them by checking corresponding boxes. The assessment is administered before and after treatment, and changes in both the overall score and items marked with high importance are analyzed. This approach allows for a comprehensive evaluation of symptom relief and improvement in quality of life (QoL). The RSOM-31 and its SNOT variants have all been recognized for their reliability in assessing sinonasal health, offering valuable insights into CRS treatment effectiveness [23-28].

The Lund-Mackay (LM) scoring system is another valuable tool for assessing treatment outcomes in those affected by CRS. This system aims to quantify the opacification and occlusion seen on computed tomography (CT) scans of CRS patients. It assigns scores to the frontal sinus, sphenoid sinus, anterior ethmoid sinus, posterior ethmoid sinus, and maxillary sinus using a scale of 0 (no abnormalities), 1 (partial opacification), or 2 (total opacification). Similarly, the ostiomeatal complex is assessed using a scale from 0 (not occluded) to 2 (occluded). Scores are determined for the left and right sides, with a cumulative score ranging from 0 to 24; a higher score indicates more severe disease. This detailed assessment of sinus pathology can be crucial for tailoring treatment plans [29]. A higher preoperative LM score may correlate to worse QoL and greater improvement in QoL post-operatively [30]. Understanding both the SNOT and LM scoring systems is crucial for interpreting the literature on treatments like FESS and OMT for CRS, as changes in overall scores provide a metric for evaluating treatment efficacy and guiding clinical decisions [25-28,30].

Functional endoscopic sinus surgery (FESS)

FESS was first introduced in 1985 to restore proper ventilation and mucociliary clearance in those with CRS or recurrent acute rhinosinusitis. The primary goal of FESS is to remove diseased or obstructive tissue, thereby improving sinus function [21]. This procedure involves the use of a fiberoptic endoscope to perform various techniques, including excising the concha bullosa, uncinectomy, maxillary antrostomy, ethmoidectomy, sphenoidotomy, and frontal sinusotomy. With over 250,000 sinus surgeries performed annually in the United States, FESS has become the gold standard for CRS unresponsive to pharmacologic therapies. It offers a less invasive and more effective alternative to traditional sinus surgery, resulting in shorter recovery times and fewer postoperative complications. Furthermore, FESS has been adapted for other pathologies, such as sinonasal tumors, pituitary tumors, and skull base defects, highlighting its versatility in addressing a range of sinonasal conditions [31,32].

Effectiveness

The reported success rate of FESS for CRS has shown considerable variability over the past few decades. Dursun et al.’s review of nine studies conducted between 1989 and 1998 found success rates of FESS ranging from 80% to 98%. However, these studies lack long-term follow-up data, which limits their external validity. To address this gap and improve upon the limitations of previous studies, Dursun et al. analyzed the success rate of FESS in 130 CRS patients in Turkey at a long-term follow-up appointment. Their retrospective analysis of patients who received surgery from January 1992 to December 1993 at a single institution found an 83% success rate at a median follow-up of 60 months. Factoring in extended follow-up assessments is believed to provide a more accurate assessment of the efficacy of FESS for CRS treatment [33].

The effectiveness of FESS is commonly evaluated by analyzing SNOT scores before and after surgery. Two studies conducted in India examined SNOT-22 scores in CRS patients who underwent FESS after pharmacologic management proved ineffective. These studies showed a mean decrease in SNOT-22 scores of 33.04 and 35 at three months after surgery [34,35]. Similarly, a study in Iran found a mean reduction of 26±8.2 in SNOT-20 scores following surgery [36]. Further corroborating the benefits of this procedure, research from Morocco demonstrated improvements in QoL across all SNOT-22 categories after FESS [37]. These findings suggest significant symptom relief and improvement in QoL for FESS patients [34-37].

In addition to the mean decrease in SNOT score observed in the Iranian study, there was a mean preoperative LM score of 18.5±5 out of 24, with a mean decrease of 8 points after surgery [36]. This indicates a reduction in disease severity through decreased opacification or occlusion [29]. However, there has been considerable debate surrounding the use of the LM staging system in predicting the efficacy of FESS. One study found significantly higher preoperative LM scores in those with CRSwNP compared to CRSsNP, while preoperative SNOT-20 scores were similar in these two groups [38]. Since patients can have differences in LM scores but similar symptomatology scores, the correlation between the two has been questioned. Nonetheless, a correlation has been found between a higher preoperative LM score and a greater decrease in postoperative SNOT scores. Therefore, those with a higher preoperative LM score may see the most benefit from FESS. These insights can help personalize treatment plans and improve patient outcomes [39].

A study by Senior et al. investigated long-term medication usage in patients with CRS who underwent FESS. The study followed 70 patients at 1.5 years and 7.8 years postoperatively. It was found that 62% of patients reported reduced antibiotic usage at 1.5 years, increasing to 82% at 7.8 years. A similar trend, though less pronounced, was observed in oral steroid and intranasal steroid use. The authors also noted a significant decrease in the duration of antibiotic treatments, from 21 weeks preoperatively to 6.1 weeks after 7.8 years [40]. Another study examining medication usage after revision FESS in patients with refractory CRS found a comparable result of less antibiotic usage, though a significant decrease in steroid or nonsedating antihistamine usage was not observed [41]. Overall, the literature suggests that the majority of patients who undergo FESS experience improved quality of life, decreased SNOT and LM scores, and a decreased need for antibiotic medication [33-41].

Revision

When initial surgery is ineffective, some patients opt to have a revision procedure. The rate of these revisions does not necessarily indicate the true percentage of patients who had ineffective FESS, as some may decide not to undergo repeat surgery. Stein et al. analyzed data from California’s State Ambulatory Surgery Database from 2005 to 2011, identifying 61,339 CRS patients who had FESS. They found that 4,078 (6.65%) returned for revision surgery during the study period. They also reported a higher revision rate among females and patients with CRSwNP [42]. However, this study may underestimate the long-term revision rate. Smith et al. used the Utah Population Database to determine a revision rate of 15.9% from 1996 to 2016, with a mean follow-up of 9.7 years. Among those who underwent revision surgery, the average interval between the first and second procedures was 4.39 years, with the time decreasing for each subsequent revision. They identified similar risk factors for revision surgery, including female sex and the presence of nasal polyps, along with additional factors such as older age at first surgery, a family history of CRS, comorbid asthma, and allergies. Recognizing these risk factors can help clinicians guide their patients in making more informed decisions regarding the treatment of CRS, potentially improving long-term outcomes [43].

Complications

Due to the proximity of critical structures surrounding the sinuses, major complications can occur with FESS. The most common major complications include orbital or eye injury, skull base injury resulting in cerebrospinal fluid (CSF) leaks or meningitis, and epistaxis. These complications can significantly impact patient outcomes and may necessitate additional interventions. Among these, CSF leaks and orbital or eye injuries are particularly concerning for both patients and surgeons [31,44]. Generally, complications from FESS are rare, and reported incidence rates vary depending on the cohort examined, the time period of the study, and what complications examiners classify as major or minor [45]. Awareness of potential complications is essential for both preoperative planning and obtaining informed patient consent.

A retrospective review of 78,944 patients who underwent FESS in California and Florida from 2005 to 2008 reported a major complication rate of 0.36% in primary FESS patients and 0.46% in revision FESS patients. Although complications from revision surgery are expected to be higher due to altered anatomy from previous surgery, the authors found both rates to be lower than previous estimates [45,46]. The authors also found that patients who had image-guided surgery had a 60% increased likelihood of developing complications. They explained that this could be attributed to the technology being used in more complex cases that inherently carry a higher risk of complications, or to a potential false sense of security that the technology provides surgeons [45].

Ramakrishnan et al. reviewed a nationwide database of 62,823 patients who underwent FESS between 2003 and 2007 and found that the rate of major complications was slightly higher at 1%. This included 0.17% of patients experiencing CSF leaks, 0.07% sustaining orbital injuries, and 0.76% having a hemorrhage requiring transfusion. The authors noted that the rate of major complications has decreased compared to reports from over a decade earlier [47].

A retrospective study by Chou et al. of 997 CRS patients in a hospital in Taiwan, conducted between 2006 and 2010, reported a similar major complication rate of 0.5%, with a minor complication rate of 7.3%. The study found that the complication rate is associated with an advanced LM score between 19 and 24, an inexperienced surgeon, and the presence of a mesenteric type of anterior ethmoid artery [48].

Osteopathic manipulative treatment (OMT)

OMT is an integral part of the holistic approach osteopathic physicians take in managing patient health. Once a physician diagnoses a patient with somatic dysfunction, a variety of techniques may be utilized to aid in the relief of symptoms. OMT is used to diagnose, prevent, and treat a broad spectrum of illnesses and injuries. This treatment modality encompasses forty hands-on techniques, ranging from gentle pressure to powerful thrusts, aiming to improve physiologic function and restore homeostasis [22,49]. These manual treatments are frequently employed to alleviate pain, such as headaches, neck pain, and back pain, by addressing structural and functional abnormalities. In addition to musculoskeletal complaints, these techniques are used to manage sinusitis and other respiratory conditions. In fact, sinusitis has been ranked among the top fifteen conditions treated with OMT [50].

Effectiveness

Lee-Wong et al. conducted a study involving the use of OMT on 15 patients for their CRS as an adjunct to their office visits. The OMT regimen included five techniques: four involving direct pressure and one involving sinus drainage. These techniques were selected to increase lymphatic flow and unblock nasal passages to decrease sinus pain, pressure, and congestion. Each technique was administered in six cycles, with each cycle lasting three minutes, totaling an 18-minute treatment. The success of the treatment was then analyzed by having the patients report both the pain associated with the treatment and the immediate effects it had on their CRS symptoms. Of the patients, 11 reported no pain during the treatment, while four reported minimal pain. The average patient-reported score for sinus pain, pressure, and congestion decreased from 3.07 out of 4 before treatment to 2.33 after. Despite the result suggesting a potential benefit, this change is not statistically significant, and further research is required to validate the effectiveness of the treatment. However, all 15 patients left the medical office reporting a reduction in pain and greater satisfaction with their visit compared to previous visits. The authors acknowledge that the absence of a control group for sham treatment represents a major limitation of the study [51].

Lee et al. intentionally built on the work of Lee-Wong et al. by including a slightly larger sample size of 22 subjects and developing a specific OMT protocol for sinusitis. They hoped that developing a protocol for sinusitis would guide future physicians in their approach to treatment. Their study employed the following techniques, with a total treatment duration of approximately 14 minutes per subject: cervical soft tissue manipulation, cervical myofascial release, cervical high-velocity/low-amplitude thrusts, thoracic muscle energy, scapular release, thoracic high-velocity/low-amplitude thrusts, Chapman points, and intra-oral sphenopalatine ganglion release. The 22 subjects participating in the study were all osteopathic medical students, with a median age of 25 years. Of these subjects, 10 had sinusitis secondary to an upper respiratory tract infection, seven had CRS, four had allergies, and one had an unknown ailment. Twelve patients were receiving pharmacologic treatments, while 10 were not. Post-treatment assessments indicated that all subjects had improvement in their nasal congestion, sinus pain, postnasal drip, headache, loss of smell, and fatigue compared to pre-treatment levels. The results showed varying levels of treatment effectiveness, with four subjects reporting minimal improvement, 16 reporting moderate improvement, and two reporting complete improvement. The authors did not specify the level of improvement in patients undergoing pharmacologic therapy, nor did they indicate whether these patients required continued medication after OMT. These authors also admitted to limitations, including the lack of a control group, small sample size, and confounding variables such as concomitant treatment, decreasing the reliability of their data. Despite these limitations, it was concluded that there is potential to effectively treat CRS with the developed treatment protocol [52].

The use of manual therapy has been identified as a potentially effective treatment for craniofacial pain in those with CRS. In one study, 14 CRS patients received manual therapy three times over a seven-week period. By the end of the treatment, all participants reported a reduction in craniofacial pain and overall severity of CRS symptoms. These findings suggest that manual therapy may be beneficial for those with craniofacial pain from CRS. Although this study utilized manual therapy administered by a physical therapist rather than OMT administered by an osteopathic physician, the authors compared their findings to a study in which OMT was performed, due to the similarities in physical manipulation methods. The authors acknowledged the limitations of a small sample size and lack of a control group and highlighted the need for studies examining medium- and long-term effects of manual therapy in CRS patients [53].

A case report published in 2018 documented the change in SNOT-22 score for a 45-year-old female patient who received OMT for CRS. The patient self-referred for complaints of facial pain, congestion, hyposmia, and nasal drip, among other symptoms, and she received OMT. The treatment regimen took place twice a week for four weeks and included counterstrain of the maxillary tender point, a milking technique of the maxillary sinus, and an intraoral sphenopalatine ganglion decongestion technique. The author found that the patient had significant improvement in her SNOT-22 score after treatment. The case report concluded that OMT may be a conservative treatment option for CRS, especially due to the lack of side effects from the treatment. Since the findings of the case study lack generalizability for a broader population, the author called for randomized clinical trials with larger sample sizes [54].

One author reviewed relevant data on the use of OMT for CRS and created a study design to address the current limitations in the literature. This design calls for a larger sample size of 48, with three groups of 16. The first and second groups would be experimental, while the third group would be a control receiving a sham treatment. The design dictates that the first group would receive a single frontal lift technique, while the second group would receive a combination of techniques like those performed by Lee-Wong et al. Participants would be treated once a week for six weeks and take the SNOT-22 prior to each treatment. They would also use the Numeric Pain Rating Scale before and after each treatment. To eliminate confounding variables present in the current literature, patients would be excluded from the study if they are using prescription medication for their CRS. This design aims to evaluate the efficacy of a single OMT technique versus a combination of techniques and to produce more reliable data by using a larger sample size and a control group [55]. A summary of studies utilizing OMT as a treatment for CRS is shown in Table 1.

**Table 1:** Findings and conclusions of studies utilizing OMT or manual therapy on patients with CRS. CRS: chronic rhinosinusitis, OMT: osteopathic manipulative treatment, SNOT-22: sinonasal outcome test-22.

References	Country	Findings	Conclusion
Lee-Wong et al. [51]	United States	The authors utilized 5 treatment techniques for 15 CRS patients. Eleven patients reported no pain during the treatment, while four reported minimal pain. The average patient-reported score for sinus pain, pressure, and congestion decreased from 3.07 out of 4 before treatment to 2.33 after it. All 15 patients reported a reduction in pain.	The study concluded that OMT may help with CRS symptoms, but the results were not statistically significant. They also concluded that OMT as an adjunct to the office visit results in greater patient satisfaction.
Lee et al. [52]	United States	The authors used eight different OMT techniques to treat 22 sinusitis patients; Seven of these patients had CRS. After treatment, four patients had minimal improvement of their symptoms, 16 had moderate improvement, and two had complete improvement.	The authors concluded that their OMT protocol could reduce sinusitis symptoms since all patients had improvement in their symptoms compared to pre-treatment.
Méndez-Sánchez et al. [53]	Spain	The authors analyzed the use of manual therapy performed by a physical therapist on 14 CRS patients with craniofacial pain. Three treatments were provided over a seven-week period, and all of the patients reported a reduction in craniofacial pain and overall CRS symptom severity at the end of the time period analyzed.	In this study, the authors concluded that manual therapy could provide patients with relief from their craniofacial pain and other symptoms of CRS. They also underscored the similarity between manual therapy techniques and OMT techniques for CRS treatment.
Baisakhiya [54]	India	In this case study, the author utilized a maxillary tender point counterstrain, a maxillary sinus milking technique, and an intraoral sphenopalatine ganglion decongestion technique on a 45-year-old female with CRS. For this patient, the treatment resulted in a significant decrease in her SNOT-22 score.	The author concluded that OMT may be a valid conservative treatment modality due to the significant decrease in symptom scores and the lack of side effects from treatment.

Limitations

The limited literature on the use of OMT for CRS suggests that it may be an effective remedy; however, these studies mostly lack control groups, have small sample sizes, and do not include long-term follow-ups, which greatly limits the reliability of the data. It is imperative to perform large-scale randomized controlled trials with extended follow-ups to validate the effectiveness of OMT as a noninterventional alternative to FESS for managing CRS.

## Conclusions

With the prevalence of CRS worldwide and its severe financial, physical, and emotional impact, it is crucial to develop a thorough understanding of available treatment options. FESS has become the gold standard for interventional treatment of CRS, due in part to its low rate of major complications compared to other interventional methods. However, when complications arise from FESS, they can significantly impact patient outcomes, often requiring additional intervention. Those at the highest risk for developing complications are patients who have had previous sinus surgery. Additionally, while FESS has been shown to improve the mean quality of life for patients with CRS, there is a risk of requiring revision surgery due to persistent disease. Patients at the highest risk of requiring revision surgery include females and those with CRS with nasal polyps. Given the possibility of complications and the need for revision surgery, it is reasonable to explore OMT as a noninterventional alternative, which may offer similar benefits without these risks. OMT is generally considered safe, and no studies have reported complications associated with its use in CRS patients. Ideal candidates for OMT may include CRS patients with an increased risk of complications, those at risk of requiring revision surgery after FESS, or patients seeking a noninterventional alternative to FESS.
